# Plant biochemical genetics in the multiomics era

**DOI:** 10.1093/jxb/erad177

**Published:** 2023-05-12

**Authors:** Saleh Alseekh, Esra Karakas, Feng Zhu, Micha Wijesingha Ahchige, Alisdair R Fernie

**Affiliations:** Max Planck Institute of Molecular Plant Physiology, 14476 Potsdam-Golm, Germany; Center of Plant Systems Biology and Biotechnology, Plovdiv, Bulgaria; Max Planck Institute of Molecular Plant Physiology, 14476 Potsdam-Golm, Germany; National R&D Center for Citrus Preservation, Key Laboratory of Horticultural Plant Biology, Ministry of Education, Huazhong Agricultural University, 430070 Wuhan, China; Max Planck Institute of Molecular Plant Physiology, 14476 Potsdam-Golm, Germany; Max Planck Institute of Molecular Plant Physiology, 14476 Potsdam-Golm, Germany; Center of Plant Systems Biology and Biotechnology, Plovdiv, Bulgaria; Boyce Thompson Institute, USA

**Keywords:** Biochemical genetics, canalization, epistasis, metabolomics, GWAS, QTL

## Abstract

Our understanding of plant biology has been revolutionized by modern genetics and biochemistry. However, biochemical genetics can be traced back to the foundation of Mendelian genetics; indeed, one of Mendel’s milestone discoveries of seven characteristics of pea plants later came to be ascribed to a mutation in a starch branching enzyme. Here, we review both current and historical strategies for the elucidation of plant metabolic pathways and the genes that encode their component enzymes and regulators. We use this historical review to discuss a range of classical genetic phenomena including epistasis, canalization, and heterosis as viewed through the lens of contemporary high-throughput data obtained via the array of approaches currently adopted in multiomics studies.

## Teaser

In this review we describe how the application of modern metabolomics techniques is providing insights into many classical genetic questions by viewing them through a biochemical lens.

## Introduction The foundation of biochemical genetics

There has been much written about Gregor Mendel in the last 12 months due to the 200th anniversary of his birth (Eckardt *et al.*, 2022). He is often described as the founder of genetics but what is less commonly acknowledged is that he is without a doubt, somewhat serendipitously, the founder of biochemical genetics. This is because of the seven characteristics of pea plants he studied, one of which was a wrinkled phenotype and was a morphological change that was displayed due to a mutation in a starch branching enzyme—a fact that was first documented more than 30 years ago (Bhattacharyya *et al.*, 1990). Until recently, biochemical genetics was centered around those metabolites that conferred highly visual phenotypes, either with respect to pigmentation, shape, or size. That said, unlike the case for Mendel, the majority of these studies followed the isolation of the first enzyme to be identified, diastase (currently known as amylase), in microbes the early 20th century (Needham, 1970; Kohler, 1972) and were also subsequent to what many regard as the foundation of biochemical genetics, namely the linking of enzymes to genes via the ‘one gene, one enzyme’ theory of (Beadle and Tatum (1941). Whilst this theory holds true (at least in part) in simple organisms such as *Neurosporra crassa* for which it was developed, the extensive gene duplication that has characterized the plant kingdom (Zhang, 2003; Fernie and Tohge, 2017), means that this is not the case for plants. Although on the one hand this presents a complication in the study of plant metabolism (and especially that of specialized metabolism), on the other hand, as we will detail below, recent studies aimed at examining metabolic gene clusters in plants have benefitted from this complexity (Zhan *et al.*, 2022). The term ‘biochemical genetics’, whilst less commonly used in plants than it was 50 years ago, refers to the combination of biochemistry and genetics. Prior to the greater availability of genome sequences over recent decades, this was largely performed by the use of molecular genetic markers and simple characteristics of gene products, for example based on their electrophoretic properties (Banuett-Bourrillon and Hague, 1979; Kawase and Sakamoto, 1984). Whilst these approaches were simple, they produced an incredible knowledge base. The marriage of genetics and biochemistry has a long history in the unravelling of plant metabolic pathways, and it has contributed to pivotal developments in phytochemistry (Somerville, 2000; Fernie, 2007), including the vital elucidation of various aspects of photosynthesis (Calvin, 1962; Kortschak *et al.*, 1965; Hatch and Slack, 1968), the discovery of transposable elements (McClintock, 1951), the establishment of the pathway of chlorophyll biosynthesis (Wolff and Price, 1957), the endosymbiont hypothesis of organelle acquisition (Gray *et al.*, 1999), and the high-level structural resolution of photosystems I and II (Zouni *et al.*, 2005; El-Mohsnawy *et al.*, 2010). In contemporary terms, biochemical genetics can be explained as the genetic diversity underpinning protein function and abundance.

**Fig. 1.**
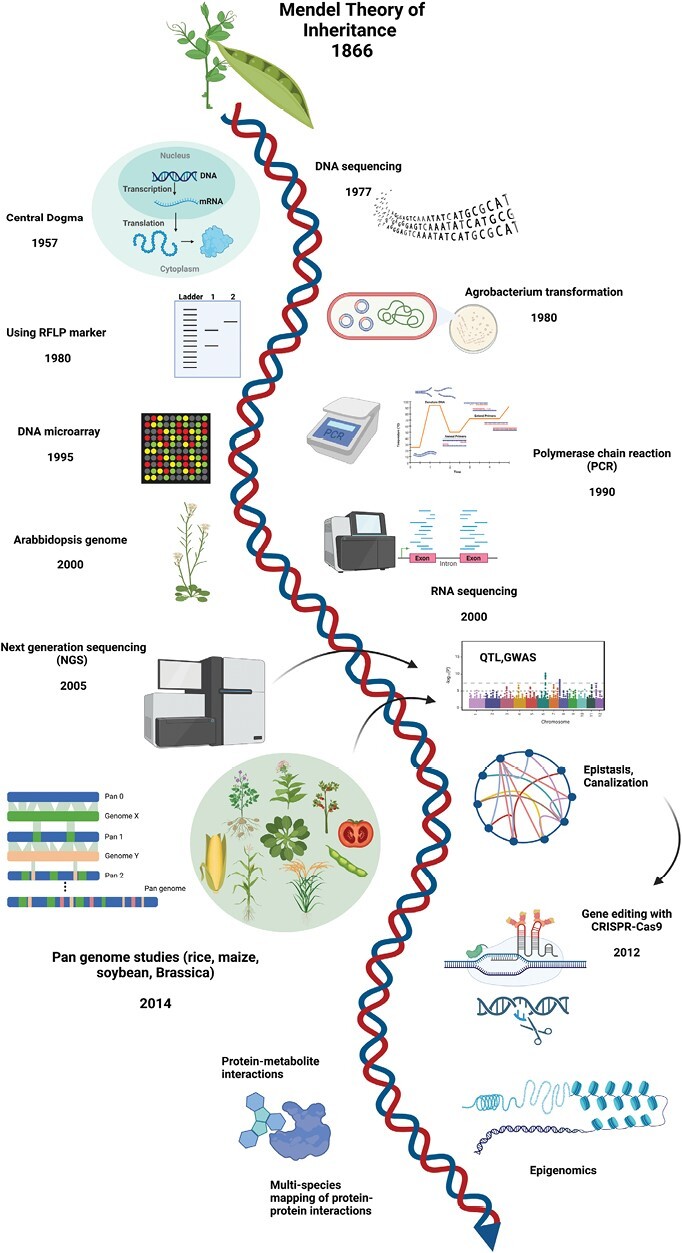
Historical developments of genetics in the multiomics era (part of the figure generated by BioRender).

Prior to the development of gene transformation and more latterly gene-editing techniques, many early studies exploited mutants in combination with either steady-state or isotope-labelled metabolite analyses (Calvin, 1962; Wheeler *et al.*, 1998), and this allowed the definition of the structures of the majority of the cardinal pathways of plant biochemistry. This approach has continued to be followed successfully in recent decades (Wheeler *et al.*, 1998; Schwender *et al.*, 2004; Tieman *et al.*, 2006; Dal Cin *et al.*, 2011). However, the dependence of metabolic studies on genetic polymorphism has also considerably diversified to include both natural variation (Kliebenstein *et al.*, 2001; Bentsink *et al.*, 2003; Schauer *et al.*, 2005; El-Lithy *et al.*, 2006) and forward- and reverse-genetic approaches (Fridman *et al.*, 2004; Sergeeva *et al.*, 2004; Schauer *et al.*, 2006; Tieman *et al.*, 2006; Zhang *et al.*, 2006; Lisec *et al.*, 2008; Rowe *et al.*, 2008; Joseph *et al.*, 2015a, 2015b). In parallel, integrated genomics approaches are beginning to yield an enhanced mechanistic understanding of biological systems, particularly when studied kinetically following an environmental perturbation or when spanning several developmental periods (Carrari *et al.*, 2006; Wang *et al.*, 2014; Yang *et al.*, 2022). In this paper, we review current and historical strategies for the elucidation of plant metabolic pathways and the genes that encode them. We additionally discuss a range of classical genetic phenomena including mode of inheritance, epistasis, canalization, and heterosis as viewed through the lens of the high-throughput data obtained via the array of approaches that are currently adopted in multiomics studies (Fig. 1). First, however, we describe the gradual shift away from using single-mutants to scanning natural variance in the wild, and discuss the difficulties and benefits of this transition.

## Single-mutants to segregating populations to genome-wide association studies: an overview of population types underpinning the study of plant biochemical genetics

As was the case noted above for *N. crassa*, the earliest reports of biochemical genetics tended to focus on mutant screens targeting single genes. Indeed, much of our understanding of the biosynthesis of starch, lipids, cell walls, and flavonols has come from screens of populations mutagenized using ethyl methanesulfonate (Herlihy *et al.*, 2019). In the case of starch metabolism, these screens were based on simple iodine staining that highlights differences in amylose binding (Caspar *et al.*, 1985; Lin *et al.*, 1988). Similarly, flavonol biosynthesis was dissected on the basis of seed colour, since the lack of flavonols in the seed coat gives rise to a transparent testa phenotype (Appelhagen *et al.*, 2014). In contrast, mutants in lipids and cell walls were largely identified by more laborious means or in developmental screens. For example, in the case of the identification of the Arabidopsis *dgd1* mutant radiolabel tracing was employed (Dörmann *et al.*, 1999), whilst mutants of cell wall biosynthesis have often been found on the basis of cell swelling phenotypes in root tissues (Doblin *et al.*, 2002). The use of stress to exacerbate phenotypes has additionally been widely employed, such as in screens for sensitivity to UV-B (Li *et al.*, 1993; Landry *et al.*, 1995; Conklin *et al.*, 1996). Since the turn of the century the availability (at least in Arabidopsis) of a T-DNA knockout collection and database (e.g. TAIR and ABRC) have greatly simplified gene functional analysis. Beyond Arabidopsis, the development of the TILLING method as an approach to accelerate the identification of mutant loci (Till *et al.*, 2003) and the development of genotyping by sequencing (Galvão *et al.*, 2012) have greatly accelerated the identification of the genetic basis of biochemical phenotypes (Fig. 1). In parallel, the advent and adoption of techniques that can simultaneously determine the levels of hundreds of metabolites (Fiehn *et al.*, 2000; Alseekh and Fernie, 2018) alongside the opportunities afforded by next-generation sequencing (Purugganan and Jackson, 2021; Gui *et al.*, 2023) have opened up the possibility of evaluating much broader genetic variance, such as that available in advanced breeding populations (Alseekh *et al.*, 2021; Sharwood *et al.*, 2022; Bulut *et al.*, 2023) and panels of natural variants (Fang and Luo, 2019). Although such populations have been reviewed extensively elsewhere (Fernie and Klee, 2011; Wijnen and Keurentjes, 2014), we will briefly describe their development here since they are highly relevant to the following sections.

Whilst the study of natural variance (at a narrow level) proceeded that of advanced breeding populations, given that it has only been possible to provide mechanistic rather than descriptive results since the recent advent of genome-wide association analysis, we will start this discussion with the use of advanced breeding populations. There are five types of population that are worthy of discussion in the context of biochemical genetics, namely recombinant inbred lines, introgression lines, backcrossed inbred lines, double-haploids, and heterogeneous inbred families (Bulut *et al.*, 2023). Populations can be generated by multiple rounds of selfing, as in the case for recombinant inbred lines (RILS; Fernie and Klee, 2011), or by repeated backcrossing and extensive genotyping, as in the case of introgression lines (ILs) and backcrossed inbred lines (BILs). Despite their similar routes of generation, BILs are more similar to RILs in that they have a mosaic of donor and recurrent genomes rather than a single (or at least a small number) of chromosomal segment substitutions (Ofner *et al.*, 2016; Brog *et al.*, 2019). Homozygous populations can also be induced by chromosomal doubling of haploids, such as for double-haploids (von Korff *et al.*, 2004). Heterogeneous inbred families are most frequently used in order to confirm quantitative trait loci (QTL) detected in a RIL population by taking a predecessor of a RIL that remains heterozygous for the region of interest but is otherwise homozygous and selfing it, following which the heterozygous region segregates in a Mendelian manner. Such studies thus enable comparison of the trait of interest for that specific region for both parental genotypes in an isogenic background. Following similar principles, multi-parental populations have been generated to increase the allelic variance in the resultant offspring, such as NAM and CUBIC populations (Gage *et al.*, 2020; Liu *et al.*, 2020). Again, we will detail the use of these populations in biochemical genetics in the following sections.

A complementary approach to that offered by breeding populations is that of directly assessing the broad-range natural variation of traits and associating this with differences in gene sequences via genome-wide association studies (GWAS; Alseekh *et al.*, 2021). The aim of GWAS is strikingly simple, namely to detect the association between allele or genotype frequency and trait status. The first step is to select an appropriate study population, considering both the size of the population and the amount of genetic and phenotypic variance that it possesses. Given that we have recently reviewed this extensively (Alseekh *et al.*, 2021), we will not detail it here; suffice to say that suitable collections now exist for a range of model species, common and even rare crop species, and also a range of non-cultivated species. These collections have made GWAS considerably easier to conduct, even compared with just a few years ago. Given that approaches based on breeding populations and GWAS are highly complementary, we will discuss them together in the following sections.

## Epistasis

In population genetics, the total genetic variance is divided into orthogonal components attributable to additive, dominance (intralocus interactions), and epistatic variance (interlocus interactions) that depend on allele frequencies (Vitezica *et al.*, 2017). Epistasis can be defined as deviation from the additivity of the combined effect of multiple variants (Falconer, 1989). At the beginning of the 20th century, the term epistasis was first brought into the scientific community by William Bateson as meaning a non-linear interaction between two or more segregating loci with different alleles across genetic backgrounds (Causse *et al.*, 2007; Liu and Yan, 2019). Most observed genetic variance for quantitative traits is additive; however, the great influence of epistatic gene action at many loci for quantitative traits should not be ignored (Liu and Yan, 2019). In the absence of epistasis, the estimates of additive and dominance effects at a given locus are the same regardless of the genotype of another locus; however, in the presence of epistasis, a substantial contribution is provided to each of these variance components.

Multiple genetic factors and their interactions (epistasis) are the key regulators for elucidating the genetic basis of phenotypic variances, and are also implicated in gene–environment interactions. The great influence of epistasis and complicated forms of environmental effects have been neglected in studies detecting phenotypic variance. The elucidation of statistical associations between millions of genetic variants and phenotypes has become possible with the use of single-nucleotide polymorphisms (SNPs), and these have also contributed to the investigation of genotype × environment (G×E) interactions (Liu and Yan, 2019). It has been suggested that G×E interactions might have impacts in complex ways within populations (Scheiner, 1993; Juenger *et al.*, 2005).

In all studies of quantitative variation, statistical power is a central issue. Theoretical models and experimental results indicate that population bottlenecks and subdivisions expose hidden additive genetic variance selection due to epistasis. Many distinct methods are available to analyse and visualize one-, two-, or three-way epistatic interactions (Martínez *et al.*, 2018). Due to the statistical and computational complexities, most analyses are constrained to examine only pairwise interactions in order to detect epistasis (Weinreich *et al.*, 2013). the approaches mainly focus on the selection of SNPs for interactions, based on existing biological knowledge or statistical features (L. Li *et al.*, 2022). When considering biochemical pathways and gene networks, the presence of epistasis may be inevitable; however, evidence of epistasis in terms of quantitative character variation is surprisingly weak (Mackay, 2014; Labroo *et al.*, 2021). In quantitative genetics, the standard non-epistatic model is often considered to be a linear approximation of the complicated mapping from genotype to phenotype. Therefore, it is now considered normal to characterize gene interactions with non-linear functions when predicting phenotype from genetic metrics.

Over recent decades, the contribution of epistasis to quantitative trait phenotypes has been apparent in many studies, especially in GWAS and QTL analysis (Yi and Xu, 2002; Manicacci *et al.*, 2009; Würschum *et al.*, 2011; Wen *et al.*, 2015; Zhang *et al.*, 2015). GWAS have been mainly conducted based on the assessment of statistical significance in the dissection of G×E interactions (Liu and Yan, 2019). Many studies have successfully mapped QTL; however, the variations that are caused by the tens to hundreds of genes underlying genomic regions remain unidentified (Allen Orr, 2001; Maloof, 2003; Koornneef *et al.*, 2004). The increasing use of QTL mapping has been cited as the key contributor for elucidating epistasis in quantitative trait variation (Erickson *et al.*, 2004).

Dominance and epistasis play key roles in the determination of complex traits of interest. The difficulties in detecting epistasis cannot be ignored, and therefore experimental set-ups capable of full characterization are necessary to test these higher-order interactions. An ideal experimental set-up would be the generation of isogenic lines harbouring distinct allelic combinations underlying the set of genes of interest. F_2_ populations or recombinant inbred populations have been used to handle epistatic mapping (Kerwin *et al.*, 2017). A strong epistatic interaction can be detected for some metabolites in kernels in maize RIL populations (Wen *et al.*, 2015). Shang *et al.* (2016) have shown that epistasis contributes to yield heterosis in RIL and backcross populations of cotton. GWAS and epistasis studies performed using 214 soybean germplasm accessions have shown that additive and epistatic variance can explain almost half of the phenotypic variance for sudden death syndrome resistance (Zhang *et al.*, 2015).

The great influence of epistasis has been demonstrated in a wide range of crop species (Schnable and Springer, 2013; Labroo *et al.*, 2021; Monforte, 2021). For instance, in common bean significant epistasis is observed for seed yield, the number of seeds per plant, and the number of pods per plant (Moreto *et al.*, 2012). In wheat, epistatic analysis using RIL mapping populations has detected one pair of epistatic QTL for the first internode component index and three pairs for the third internode component index, thereby providing information about plant height components and associated increases in yield (Qin *et al.*, 2022). Another study of common bean has indicated the role of epistasis in the genetic control of traits associated with yield in inter-gene-pool crosses (Johnson and Gepts, 2002), and in rice it has been shown that epistasis regulates plant height on the genetic basis of midparent heterosis (Shen *et al.*, 2014). Epistatic QTL interactions including synergistic interactions have been identified in wheat populations that shed light on the inheritance of shattering resistance (Bokore *et al.*, 2022). Metabolite profiling using a maize backcross population detected ~15% epistatic interactions for primary metabolites that have impacts on maize quality, and hence could be utilized for improvement (K. Li *et al.*, 2019). Away from crop species, Kerwin *et al.*, (2017) conducted a detailed analysis that identified both additive and epistatic interactions for the polymorphic genes that control aliphatic glucosinolate in Arabidopsis that had not previously been assessed for potential interactions with the environment. In addition, crossing of C24 with Col-0 has indicated that dominance and epistatic interactions have an important role in biomass-related traits (Kusterer *et al.*, 2007).

## Canalization

**Fig. 2.**
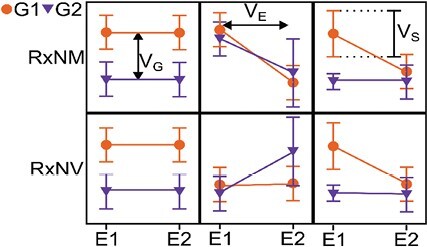
Concepts of canalization and variance. Schematic plots are shown for the mean (±SD) of theoretical traits of two different genotypes, G1 and G2, in two different environments, E1 and E2. The top row shows plots for the reaction norm of the mean (RxNM) whilst the bottom row shows plots for the corresponding variance (RxNV). The sources of variation are either due to genetic (V_G_), environmental (V_E_), or stochastic (V_S_) factors.

Much less well understood than the genetics that contribute to the level of a given quantitative trait are those that are responsible for the robustness of the trait between or within environments (Fig. 2). An early principle for the concept of robustness in a developmental context was described by Waddington (1942). Waddington noticed that developmental processes were generally canalized so as to bring about a single clearly defined final stage, regardless of minor variations in the conditions under which the processes occurred. Based on this, Waddington suggested that there must be some capacity of the genotype to buffer the phenotype against these minor variations in genotype and environment. In that sense, a distinction can be made between genetic and environmental canalization. Perhaps the best-studied developmental process that shows considerable canalization against different sources of variation is the patterning of the vulva plate of *Caenorhabditis elegans* (Félix and Barkoulas, 2012). In both *Drosophila* and in Arabidopsis, the chaperone HSP90 has been shown to be a capacitor of phenotypic variation (Rutherford and Lindquist, 1998; Queitsch *et al.*, 2002), providing another example of developmental canalization. Genes such as HSP90 are often considered to be so-called gene network hubs, where the exponential distribution of connectivity is associated with robustness (Lachowiec *et al.*, 2016).

Quantitative traits seem to have received less attention than developmental ones in terms of canalization. When considering quantitative traits of different genotypes in different environments it becomes clear that there are different forms of canalization that can be considered, and it seems reasonable and necessary to also use a terminology that works in a quantitative sense. Using the reaction norm, that is how the trait of a genotype changes between environments, it is possible to distinguish the reaction norm of the mean (RxNM) and the reaction norm of the variance (RxNV) of a trait (Dworkin, 2005). While the former considers the change of mean values of genotypes between environments, the latter considers the change of variance between individuals between environments. Accordingly, the sources of phenotypic variation can be either genetic, environmental, or stochastic factors (Fig. 2; Laitinen and Nikoloski, 2019).

While there are examples with respect to canalization of quantitatively characterized traits ranging from single-celled organisms to higher plants (Alon *et al.*, 1999; Lehner, 2010; Fisher *et al.*, 2017; Lachowiec *et al.*, 2018), the application to metabolism is relatively recent and more limited. A flux balance analysis in *E. coli* identified six capacitor reactions that have a high influence on genetic canalization of metabolism (Ho and Zhang, 2016). In Arabidopsis, the *ELF3* gene shows an effect on the canalization of both the circadian clock as well as glucosinolate levels (Jimenez-Gomez *et al.*, 2011). By using transformed data to assess the reaction norm of the variance, several canalized metabolic QTL (cmQTL) responsible for genotype × environment effects on the variance of various tomato fruit metabolites have been detected (Alseekh *et al.*, 2017). Interestingly the cmQTL only sparsely overlapped with the QTL for the levels of the metabolites, suggesting different loci were related to level and variance. Further validation of candidate genes supports the idea that genes that affect (e.g.) the cross-environment canalization do not necessarily also affect the inter-individual variance. The observation that loci responsible for variation in a trait are (at least partly) distinct and also fewer in number than loci responsible for the level of the trait is supported by other research (Hall *et al.*, 2007; Joseph *et al.*, 2015a; Li *et al.*, 2016; Kusmec *et al.*, 2017). Both the distinctiveness and the reduced number of loci could point to there being a few regulatory genes that simultaneously control several traits (Alseekh *et al.*, 2017). Depending on the trait, we can consider many different combinations of effects of loci on either the level or variance of that trait, or both (Fig. 2, columns 1, 2, and 3, respectively).

In this regard, datasets from large mapping studies that have previously been used simply to study the level of a given trait could be reused to study the inter-individual or cross-environment variation. While this has been done in some cases (Schauer *et al.*, 2006; Wentzell *et al.*, 2007; Schauer *et al.*, 2008; Jimenez-Gomez *et al.*, 2011; Alseekh *et al.*, 2017), this resource of legacy data is still underutilized. Together with new data generated through the high-throughput platforms of the multiomics era, it can be used to study canalization and variation more comprehensively (Wijesingha Ahchige *et al.*, 2023).

## Organellar inheritance of biochemical traits

The organellar inheritance of biochemical genetic traits (also known as cytoplasmic genetic variation) is a further type of epistasis that we consider separately here given that it has long been studied in its own right. Organellar genomic variation can be linked to dramatic phenotypic alterations in both mammals (Johns *et al.*, 1992; Taylor and Turnbull, 2005; Schon *et al.*, 2012) and plants (Schnable and Wise, 1998; Roubertoux *et al.*, 2003). The use of structured populations in yeast and animals have variously shown that cytoplasmic variance can influence fitness, cognition, and biomass as well as altering fitness and creating hybrid isolation (Dimitrov *et al.*, 2009; Wolf, 2009; Willett, 2012). Similarly, in plants mitochondrial genetic variation is linked to important quantitative phenotypes including cytoplasmic male sterility (Hanson, 1991; Schnable and Wise, 1998), whilst plant breeding efforts commonly employ diallele crosses to assess the presence of maternal effects on phenotypes such as height in maize (Tang *et al.*, 2013). Moreover, the presence of cytoplasmic and nuclear genome interactions has been found to influence a range of agronomic traits (Tao *et al.*, 2004); however, the genes involved are yet to be identified. Such variation is uncovered in experiments in which variance in organellar genomes is assessed alongside that of the nuclear genome. Due to their differing modes of inheritance, in practice this is largely achieved via varying the organellar genome within a population in which the nuclear genome has undergone considerable recombination. This is most simply achieved by the analysis of reciprocal F_2_ populations; however, historically in plants such analyses have been taken to imply that cytoplasmic effects on phenotypic variation are quite small (Singh, 1966; Crane and Nyquist, 1967; Eenink and Garretsen, 1980; Bhatt *et al.*, 1983; Rehal *et al.*, 2022). However, in contrast to previous estimates of small effects, genomic sequencing within Arabidopsis has shown the presence of considerable genetic polymorphism in both the plastidic and mitochondrial genomes, suggesting the potential for broad phenotypic consequences (Moison *et al.*, 2010). In an attempt to quantify the importance of cytoplasmic variance, Joseph *et al.* (2013) utilized metabolomics to investigate how genetic variation in the cytoplasmic and nuclear genomes interacts to control metabolome variation in a reciprocal Arabidopsis Kas × Tsu RIL population (Juenger *et al.*, 2006; McKay *et al.*, 2008). They found that variation in the organellar genome contributed to variation in the levels of more than 80% of the metabolites studied. Organellar genes also helped to regulate the effect of nuclear genes. This combination of direct and indirect influences helps to explain how a small number of organellar genes can have a disproportionately large effect on phenotype. However, despite the considerable insights provided by this study, it is our contention that a considerable deficit remains in our overall knowledge of the importance of these interactions, and it will be highly important to boost our understanding of their importance in crop species as well as to employ recently developed approaches (Alseekh *et al.*, 2021) to ascertain whether differences in their influence have arisen during the processes of domestication and crop improvement.

## Use of populations in metabolite and gene annotation

Plant metabolic studies have traditionally focused on the role and regulation of the enzymes that catalyse key reactions within specific pathways (Fernie and Tohge, 2017). Although metabolic regulation is still being addressed via reverse-genetics approaches such as transgenesis, and via novel molecular techniques such as genome editing, within recent years broad natural variance in the form of linkage mapping populations and GWAS have been shown to be effective tools by which to deepen our understanding of plant metabolism (Alseekh *et al.*, 2021). As mentioned above, there has been a long history of utilizing genetics to understand plant biochemistry, as demonstrated by Mendel’s early studies on peas (Mendel, 1865; White, 1917). In plant metabolism, a further seminal study was the discovery of transposable elements in maize kernels that regulate the flavonoid biosynthesis genes (McClintock, 1950). The genetic regulation of plant flower color, which is mainly an attribute of the abundance of secondary metabolites such as anthocyanins and flavonoids, has been investigated over the last two centuries (Hooker, 1837; Sink, 1973).

Sax (1923) was the first to describe QTL mapping in studying the seed size and color of common bean. Modern QTL mapping, which is defined in segregating populations, is based on the genotyping of progeny derived from a cross between distinct genotypes and the association of molecular markers with the phenotype of interest in the resultant offspring (Broman, 2001). It was facilitated by development of comprehensive DNA markers (Kumar, 1999; Phillips and Vasil, 2001) and more recently by the release of many plant genomes (Kress *et al.*, 2022). In terms of plant metabolism, early use of mapping populations focused mainly on important and easy-to-score metabolic traits such as carotenoid content in tomato, protein and oil content in maize, and starch content in potato and rice (Moose *et al.*, 2004; Fernie *et al.*, 2006). The initial QTL mapping studies using high-throughput mass spectrometry (MS) techniques and assessing the levels of multiple metabolic traits was first made in 2006. These studies looked at the primary metabolism of tomato (Schauer *et al.*, 2006) and the contents of secondary metabolites in Arabidopsis (Keurentjes *et al.*, 2006) to evaluate the natural variations in metabolism present in biparental segregating populations. They were followed up by further mapping studies in the same laboratories that addressed many aspects of the genetics of metabolism, including comparative analyses of population types and the evaluation of heterosis, heritability, and the environmental plasticity of the plant metabolome (Lisec *et al.*, 2008; Rowe *et al.*, 2008; Joseph *et al.*, 2015a, 2015b). This approach has been successfully applied to various types of segregating populations across a wide range of other important crop species, including maize (Wen *et al.*, 2015; K. Li *et al.*, 2019), rice (Matsuda *et al.*, 2012; Chen *et al.*, 2018), wheat (Hill *et al.*, 2013), barley (Zeng *et al.*, 2020), pepper (Wahyuni *et al.*, 2014), eggplant (Sulli *et al.*, 2021), and potato (Carreno-Quintero *et al.*, 2012). Examples such as these highlight the power of interspecific breeding populations for understanding the genetic bases of plant metabolism where large numbers of genes have been cloned and characterized. Although considerable biological insights have been obtained from using linkage mapping, the adoption of GWAS has allowed for the greatest breakthroughs given the advent of next-generation sequencing (Nguyen *et al.*, 2019). This approach relies on testing genetic variants across the genomes of many individuals of a population to identify genotype–phenotype associations (Uffelmann *et al.*, 2021). Similar to the linkage-mapping approach, GWAS has been successfully combined with metabolomics to assess the genetic bases of natural variance in plant metabolomes. This has been extensively reviewed elsewhere (Luo, 2015; Fang and Luo, 2019; Alseekh *et al.*, 2021), and here we will just highlight some examples using this approach that have identified effects of genetic variants on metabolic diversity across natural populations. GWAS in plants was initially applied to Arabidopsis (Chan *et al.*, 2010, 2011; Angelovici *et al.*, 2013; Verslues *et al.*, 2014; Strauch *et al.*, 2015) and then successfully extended to several crop species, covering primary and quality-related metabolites such as in tomato (Sauvage *et al.*, 2014; Tieman *et al.*, 2017; Ye *et al.*, 2017), wheat (Rathan *et al.*, 2022), jujube (Hou *et al.*, 2020), cassava (Rabbi *et al.*, 2022), peach (Y. Li *et al.*, 2019), and apple (Liao *et al.*, 2021). Similar approaches have been applied for specialized secondary metabolites in rice (Dong *et al.*, 2015; Matsuda *et al.*, 2015; Yamamura *et al.*, 2015; Chen *et al.*, 2016; Zhu *et al.*, 2018), maize (Wen *et al.*, 2014; Zhou *et al.*, 2019), wheat (Chen *et al.*, 2020), cucumber (Zhou *et al.*, 2016), and lettuce (Zhang *et al.*, 2020). Whilst many genes involved in plant metabolism pathways have been identified and cloned through large-scale GWAS, there are some examples of smaller-scale studies achieving good results. For example, the pathways of saiginols (phenylacylated flavonols) (Tohge *et al*., 2016) and dolichols in Arabidopsis (Gawarecka *et al.*, 2022), lignin precursors in Arabidopsis, maize, and poplar (Vanholme *et al.*, 2019), and steroidal glycoalkaloids in solanaceous species (Schwahn *et al.*, 2014) have all been refined by comparisons of only a handful up to around 100 genotypes. In principal, mapping of an unknown compound to a gene gives hints as to the structure of that compound, especially in cases where the gene encodes an enzyme involved in the biosynthesis pathway. Indeed, application of cross-over theory can be employed to check for corresponding changes in the substrate(s) or product(s) of the reaction catalysed as an additional means of validation. Even if the associated gene is a regulatory rather than a structural one, the vast array of available co-expression datasets (Mutwil *et al.*, 2011) alongside the massive recent increases in our understanding of the functions of transcription factors (see for example Tang *et al.*, 2021) means that mapping these differences to the respective metabolic pathways is becoming easier.

## Uncovering linkages from metabolites to morphology

As noted above, the linkage between morphological and metabolic phenotypes dates back to the foundation of genetics, even if Mendel himself was not aware of this. Much of the early study of biochemical genetics directly addressed these links since the majority of early genetic screens were based on morphological or other visual phenotypes. Following the development of genetic transformation protocols, however, much information was gleaned by the overexpression, down-regulation, and systematic knock out of genes (Stitt and Sonnewald, 1995; Alonso and Ecker, 2006). Whilst knockout mutants are random in nature, modern techniques now allow precisely targeted studies with genes essentially being knocked out to order. Such studies have now provided (almost) saturation coverage of the major of plant primary metabolic pathways, including the Calvin–Benson, tricarboxylic acid (TCA), and photorespiratory C_2_ cycles, as well as gluconeogenesis, the sucrose-to-starch transition, and amino acid metabolism (Galili *et al.*, 2016; Fernie and Bauwe, 2020; Fernie *et al.*, 2020; Walker *et al.*, 2021; Rosado-Souza *et al.*, 2022). Since this body of work has been comprehensively reviewed elsewhere, we will not cover it in detail here; suffice to say that it has provided a vast number of targets for breeding and that many of the effects of the genetic interventions have now been verified as resulting in improved performance in the field as well as in the laboratory. In keeping with the theme of this historical review, we will instead focus the rest of this section on a classical theme of genetics, namely heterosis. In their review on this phenomenon 16 years ago, Lippman and Zamir (2007) described it a plant biological mystery that has endured since Charles Darwin famously described how hybrids display superior growth and fertility to their parents (Darwin, 1876). Such hybrid vigor was rediscovered in maize breeding some 30 years later (East, 1908; Shull, 1908), and it was subsequently demonstrated to occur in many other crop species. Indeed, it is now widely used in agriculture with in excess of 65% of worldwide maize, sorghum, and sunflower production being hybrid-based. Yield gains of 15–50% cited by Lippman and Zamir (2007) have provided a great incentive to understand the underlying mechanisms, and y*et al*though we have made great inroads into understanding the genetic basis of yield heterosis, most notably in tomato (Lippman and Tanksley, 2001; Krieger *et al.*, 2010; MacAlister *et al.*, 2012; Park *et al.*, 2012; Jiang *et al.*, 2013; Soyk *et al.*, 2017; Torgeman and Zamir, 2023) and maize (Lai *et al.*, 2010; Yang *et al.*, 2017; Birdseye *et al.*, 2021; C. Li *et al.*, 2022), a detailed understanding of the molecular mechanisms and how they are integrated with metabolism remains lacking. There are excellent reviews available concerning the genetics underpinning heterosis (e.g. Lippman and Zamir, 2007), and so we will focus here on studies evaluating metabolic events underpinning growth and yield that have been elucidated via examination of either restricted or broad (natural) genetic variance. Our first example is provided by the assessment of what are now very well characterized *Solanum penellii* introgression lines that contain individual chromosomal segmental substitutions of the donor species in the background of the cultivated tomato *S. lycopersicum* (Alseekh *et al.*, 2013). In their original approach, Schauer and co-workers utilized a then-novel metabolic profiling protocol to evaluate the contents of primary metabolites and uncovered a large number of QTL, and they also demonstrated clear links (mainly negative ones) between metabolite levels and traits associated with yield (Roessner *et al.*, 2001). In a follow-up study, Schauer *et al.* (2008) found that these links were not present when the introgression lines were in the heterozygous state, suggesting that metabolite contents could be enhanced following this approach in the absence of a yield penalty. At around the same time, the group of Thomas Altmann used the same approach to characterize metabolite levels and growth in Arabidopsis Col-0 × C24 introgression lines and using statistical methods they were able to detect a metabolic signature for growth (Meyer *et al.*, 2007). In follow-up studies in collaboration with the group of Albrecht Melchinger, they were able to apply the same approach to the growth of maize, including the use of metabolomic prediction of growth phenotypes (Riedelsheimer *et al.*, 2012). Importantly, further studies demonstrated the utility of this approach in showing that metabolite profiling of young leaves could predict biomass at harvest (Vergara-Diaz *et al.*, 2020), and similar findings have also been published for wheat and canola (Knoch *et al.*, 2021), and also with regard to predicting responses to stress (Villate *et al.*, 2021). Furthermore, this approach is not only useful for predicting yield, as recent work has illustrated its potential use in predicting properties related to fruit flavour (Colantonio *et al.*, 2022). The growing trend of characterizing large populations at the multiomics level offers yet further opportunities for us to obtain more complete insights into both the mysteries of heterosis *per se* and the linkages between metabolism and growth and development in general.

## The metabolic basis of plant immunity

During their lifespan, plants are threatened by various different biotic challenges, including those posed by bacteria, fungi, and insects. To overcome these challenges, plants have evolved two layers of immunity response to pathogens, namely pathogen-associated molecular pattern (PAMP)-triggered immunity (PTI) and effector-triggered immunity (ETI), which require the function of different metabolites (Zeier, 2013; Piasecka *et al.*, 2015; Yuan *et al.*, 2021). The waxes and lignin of plant surfaces represent the first structural barriers for pathogens, and after passing these physical barriers, PAMPs of the microbes, which include bacterial proteins and endotoxins and the fungal cell-wall component chitin, are recognized by specific pattern-recognition receptors (PRRs), thereby activating various PTI defense responses (DeFalco and Zipfel, 2021). Among the PAMPs, chitin (an insoluble polymer of β-1,4-linked *N*-acetylglucosamine) has long been well known and hence been the subject of a large amount of study. Chitinases secreted by host plants can hydrolyse the insoluble polymer to chitin oligosaccharides, which can then be recognised by plasma membrane-localized lysin-motif (LysM) proteins, and this in turn induces the accumulation of antimicrobial metabolites such as reactive oxygen species (ROS) and salicylic acid (SA) (Gong *et al.*, 2020). In an evolutionary arms race, pathogens developed specialized effector proteins to suppress PTI, in response to which plants evolved R-proteins to recognize the active effectors, thereby inducing ETI responses and leading to the activation of mitogen-activated protein kinase signalling pathways and the promotion of more SA accumulation (Tsuda *et al.*, 2013; Shinya *et al.*, 2015). In Arabidopsis, SA can induce the folding and docking of the SA-binding domain to the ankyrin repeats of NONEXPRESSOR OF PATHOGENESIS-RELATED GENES 1 (NPR1), which affects its activity as a transcriptional cofactor and activates the expression of NPR1-dependent systemic acquired resistance genes (Kumar *et al.*, 2022). In addition, another immunity-related hormone, jasmonic acid (JA) induces a different type of systemic resistance called induced systemic resistance (ISR), and studies have indicated that SA and JA immune signaling can interact with each other to balance plant responses against pathogens (Ton *et al.*, 2002; Hou and Tsuda, 2022).

In addition to functioning in pathogen recognition, immunity signalling, and response regulation, some primary metabolites can participate in the immunity process by acting as precursors of key immunity metabolites, and some secondary metabolites can act directly as antimicrobial metabolites (Hartmann and Zeier, 2018; Huang *et al.*, 2021). For example, the important amino acid lysine can be catalysed by AGD2-like defense response protein 1 and SAR-deficient 4 to synthesize pipecolic acid, which is then transferred to a cyclic, non-protein amino acid, *N*-hydroxypipecolic acid (NHP), catalysed by flavin-dependent-monooxygenase 1 (Návarová *et al.*, 2012; Hartmann *et al.*, 2018). Recently, NHP has been demonstrated to induce the SA-independent signal pathway of plant innate immunity and systemic acquired resistance (Holmes *et al.*, 2021; Mohnike *et al.*, 2021). Putrescine, the most abundant polyamine, is a product of ornithine and arginine catabolism and it can induce callose deposition and the expression of several PTI marker genes through the hydrogen peroxide and NADPH oxidase (RbohD and RbohF) signaling pathway (Liu *et al.*, 2019; Gerlin *et al.*, 2021). Another polyamine, spermine, can inhibit the ROS burst and the associated sharp increase in the cytosolic Ca^2+^ concentration, and it acts as the repressor of the earliest signaling events of PTI and downstream transcriptional and metabolic immunity responses (Zhang *et al.*, 2023). Plant secondary metabolites have been demonstrated to possess high bioactivity that efficiently controls both herbivores and pathogens (Zaynab *et al.*, 2018). Based on an integrative analysis of the genetic screening of a 26-parent recombinant inbred line population with unbiased transcriptomic and MS-based metabolomic analyses, the novel compound scaffeoylputrescine-5-(*Z*)-3-hexenal has been identified as playing a powerful role in non-host resistance against *Empoasca* leafhoppers (Bai *et al.*, 2022). In recent decades, many studies have reported that phenylpropanoid metabolism plays important roles in resistance to both abiotic stress and pathogen attack (Tohge *et al.*, 2016; Huang *et al.*, 2021). A combination of transcriptomics, metabolomics, and sensitivity assays of 580 tomato lines to *Botrytis cinerea* has shown that the predominant tomato flavonoid naringenin chalcone together with two flavonoid glycosides (3ʹ,5ʹ-di-*C*-glucosylphloretin and phloretin-trihexose) are representative fungal resistance-related metabolites (Szymański *et al.*, 2020).

In recent decades, climate change has not only caused abnormal rainfall patterns and temperatures that induce drought and heat stress on plants, it has also changed the habits of temperature-dependent pathogens and increased the risk of infection of plants (Chaloner *et al.*, 2021). It is therefore necessary to enhance efforts to comprehensively elucidate the metabolomic basis of plant immunity. We envisage that such efforts will be accelerated by the development of efficient treatments to overcome plant microbes and to supply novel metabolic engineering genes and pathways to improve plant biotic stress tolerance. Before this is possible, however, a considerably higher number of multiomics studies of the effects of a given pathogen on a given crop need to be carried out. The study by Liu *et al.* (2018) of the role of OsWRKY67 in regulating responses to rice leaf panicle blast and bacterial blight diseases represents an interesting example of such a study, but we will need to examine far more pathogen–host responses in order to gather information regarding the commonalities and specificities of defense responses. As for understanding metabolite accumulation *per se*, utilizing broad natural variance as well as advanced breeding lines will probably represent invaluable tools to address this question in the coming decades.

## The next 100 years

In the sections above, we have documented how the answers to many classical questions in genetic have either influenced, or been influenced by, biochemical factors. One notable omission from our review is the subject of the impact of epigenetics and epigenomics on metabolism and vice versa. There were two reasons for this: firstly, two recent reviews by Samo *et al.* (2021) and Hayashi *et al.* (2023) have covered this subject, the latter in a highly comprehensive manner, and secondly, we agree with the contention of (Weigel, 2016) that the majority of the influence is retained in the genome *per se*. This fact notwithstanding, the study of epigenetic and epigenomic control of biochemical traits is clearly an area in which we can anticipate considerable breakthroughs in the future. Another area of great promise is the prospect of gaining a far more comprehensive understanding of gene–environment interactions that encompasses both canalization and plasticity, with the genetic populations and analytical tools required to address these topics now becoming increasingly available and well tested (Alseekh *et al.*, 2017; N. Liu *et al.*, 2021), as is the mathematical framework by which to assess them (Alseekh *et al.*, 2017; Laitinen and Nikoloski, 2019). The adaptation of such approaches should ultimately allow the development of truly sustainable agriculture (Fernie and Sonnewald, 2021; J. Liu *et al.*, 2021), which is arguably the greatest challenge for humanity of the 21st century.
